# Global investigation of estrogen-responsive genes regulating lipid metabolism in the liver of laying hens

**DOI:** 10.1186/s12864-021-07679-y

**Published:** 2021-06-09

**Authors:** Junxiao Ren, Weihua Tian, Keren Jiang, Zhang Wang, Dandan Wang, Zhuanjian Li, Fengbin Yan, Yanbin Wang, Yadong Tian, Kepeng Ou, Hongjun Wang, Xiangtao Kang, Hong Li, Xiaojun Liu

**Affiliations:** 1 College of Animal Science and Veterinary Medicine, Henan Agricultural University, Zhengzhou, 450002 China; 2 Henan Innovative Engineering Research Center of Poultry Germplasm Resource, Henan Agricultural University, Zhengzhou, 450002 China; 3 International Joint Research Laboratory for Poultry Breeding of Henan, Henan Agricultural University, Zhengzhou, 450002 China; 4 College of Pharmacy, National and Local Joint Engineering Research Center of Targeted and Innovative Therapeutics, Chongqing Key Laboratory of Kinase Modulators as Innovative Medicine, Chongqing University of Arts and Sciences, Chongqing, China; 5 Center for Cellular Therapy, Medical University of South Carolina, Charleston, SC 29425 USA

**Keywords:** RNA-Seq, ChIP-Seq, miR-144, estrogen, Chicken, Liver

## Abstract

**Background:**

Estrogen plays an essential role in female development and reproductive function. In chickens, estrogen is critical for lipid metabolism in the liver. The regulatory molecular network of estrogen in chicken liver is poorly understood. To identify estrogen-responsive genes and estrogen functional sites on a genome-wide scale, we determined expression profiles of mRNAs, lncRNAs, and miRNAs in estrogen-treated ((17β-estradiol)) and control chicken livers using RNA-Sequencing (RNA-Seq) and studied the estrogen receptor α binding sites by ChIP-Sequencing (ChIP-Seq).

**Results:**

We identified a total of 990 estrogen-responsive genes, including 962 protein-coding genes, 11 miRNAs, and 17 lncRNAs. Functional enrichment analyses showed that the estrogen-responsive genes were highly enriched in lipid metabolism and biological processes. Integrated analysis of the data of RNA-Seq and ChIP-Seq, identified 191 genes directly targeted by estrogen, including 185 protein-coding genes, 4 miRNAs, and 2 lncRNAs. In vivo and in vitro experiments showed that estrogen decreased the mRNA expression of PPARGC1B, which had been reported to be linked with lipid metabolism, by directly increasing the expression of miR-144-3p.

**Conclusions:**

These results increase our understanding of the functional network of estrogen in chicken liver and also reveal aspects of the molecular mechanism of estrogen-related lipid metabolism.

**Supplementary Information:**

The online version contains supplementary material available at 10.1186/s12864-021-07679-y.

## Background

Liver is the central organ of lipid metabolism, especially in chicken, where more than 90% of the de novo synthesis of fatty acids takes place [1–3]. During egg-laying periods, large amounts of triacylglycerols, cholesteryl esters, and free fatty acids are synthesized and assembled into egg-yolk precursors such as very low-density lipoprotein (VLDL) and vitellogenin (VTG) in the liver, then secreted into the blood stream, transported to the ovary and taken up by the growing oocytes [4]. Our previous RNA-Seq analysis on the liver of pre-laying (20 weeks old) and peak-laying chickens (30 weeks old) identified 960 differentially expressed (DE) genes including mRNA and non-coding RNA genes, many of which have been shown to participate in lipid metabolism pathways [5, 6]. Hence, the active lipid related genes in liver plays an important role in adaption of the physiological changes from pre-laying to peak-laying stages in hens.

The physiological processes of lipid metabolism in chicken liver are tightly regulated by estrogen at the egg-laying cycle [7, 8]. Estrogen exerts essential physiological functions mainly through the two known nuclear estrogen receptor (ER) subtypes ERα and ERβ, which directly bind to the consensus estrogen response elements (EREs) [9, 10] or indirectly bind to nuclear DNA by interacting with other DNA-bound transcription factors such as Ap1and SP1 [11, 12] to regulate transcription of their target genes. In addition, estrogen can also specifically bind to the G protein-coupled receptor GPER (known as GPR30) and activate the intracellular signaling cascade [13]. All of the receptors may synergistically or antagonistically mediate multiple physiological and pathological processes by the interaction of all activation and inhibition pathways [14]. To further understand the molecular mechanism underlying the complex interactions among transduction pathways in response to estrogens, global maps of ER binding sites and target genes in the whole genome were obtained in various cell types and tissues by employing chromatin immunoprecipitation (ChIP)-based assays [9, 15, 16]. However, only some classic estrogen target genes such as apolipoprotein B (APOB) and apovitellenin 1 (APOV1) are reported to be regulated by estrogen in chicken [17, 18]. Therefore, systematic study on the estrogen-responsive genes including mRNAs and noncoding RNAs is necessary for revealing the regulatory mechanism of lipid metabolism in chicken liver.

In this study, RNA-seq was performed to investigate the expression profiles of mRNAs, lncRNAs, and miRNAs in livers of estrogen-stimulated and control juvenile hens. ChIP-Seq of ERα was conducted to map estrogen receptor binding sites in liver. Integrative analysis of the above RNA-seq data and ChIP-seq data was carried out to explore the genome-wide estrogen-responsive genes involved in lipid metabolism mediated via ERα in the chicken liver. One of the estrogen-mediated lipid metabolic pathways regulated by miR-144 was proven. The results provide a genome-wide comprehensive expression profile of mRNAs and noncoding RNAs directly regulated by estrogen via ERα-binding sites and the estrogen-regulated network of lipid metabolism in chicken liver.

## Results

### Effects of estrogen on lipid metabolism in chicken liver

**Fig. 1:**
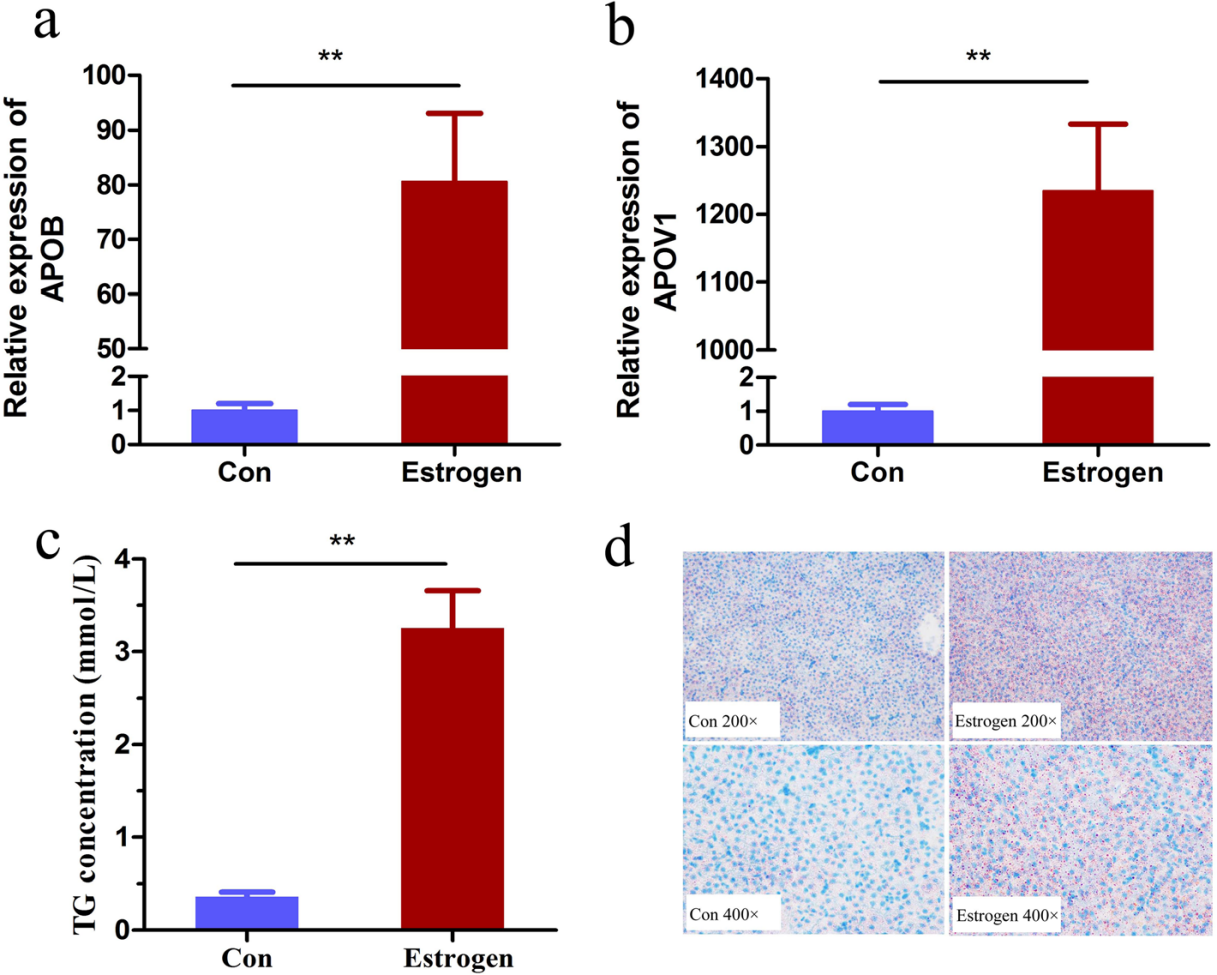
Effect of 17β-estradiol on lipid metabolism in chicken. **a** qRT-PCR analysis of liver *APOB* mRNA induction in response to 17β-estradiol. **b** qRT-PCR analysis of liver *APOV1* mRNA induction in response to 17β-estradiol. **c** Assessment of plasma TG upregulation in response to 17β-estradiol. **d** A liver tissue section stained with oil red O and hematoxylin. All error bars represent SEM, *n* = 6 for the control group and *n* = 8 for the 17β-estradiol treatment group, **p < 0.01. Chicken injected with 17β-estradiol at 8.0 mg / kg of body weight were used in this part

To verify whether exogenous estrogen had a biological role in chicken, the mRNA expression levels of the two classic target genes of estrogen, *APOB* and *APOV1,* were detected in liver tissue. The expression of APOB mRNA was significantly increased 80-fold, and APOV1 increased 1250-fold in the estrogen stimulated group compared to the control group (Fig. 1a, b). This indicated that estrogen administration was effective. The triglyceride (TG) content in the serum was significantly increased after estrogen treatment (*P < 0.05*) (Fig. 1c). The result of oil red O staining of liver tissue showed more lipid droplets in the liver of estrogen-treated chicken than in the controls (Fig. 1d). These data suggest that estrogen stimulation can significantly promote lipid synthesis and transport in the chicken liver.

### Identification of estrogen-responsive mRNA and ncRNA in chicken liver

**Fig. 2:**
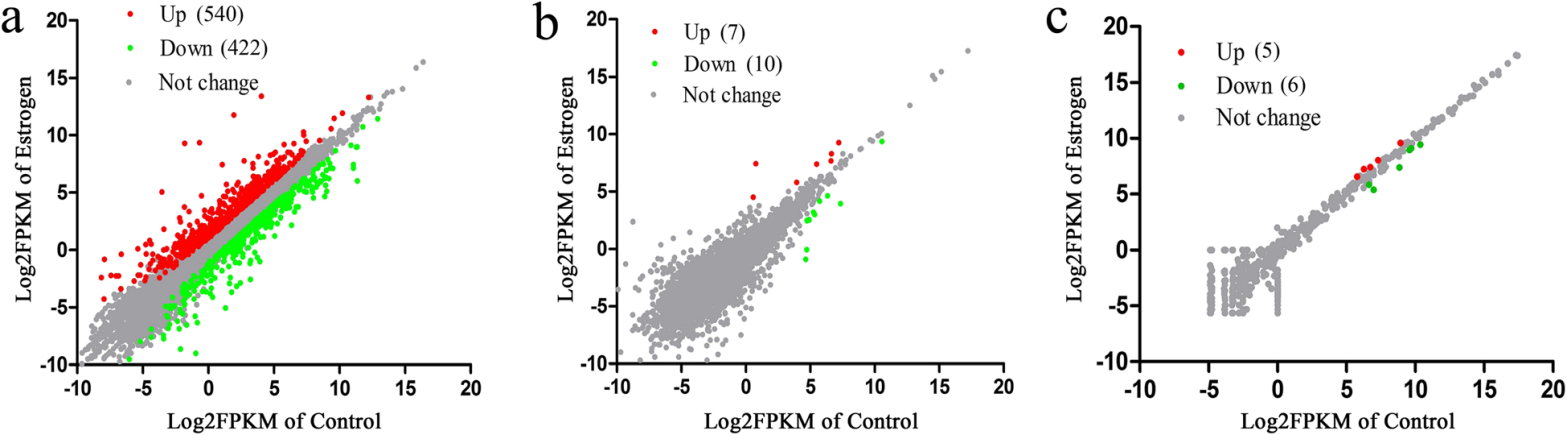
RNA-Seq data analyses. **a**, **b** The Log_2_^FPKM^ scatter plot depicting changes in the expression of protein-coding mRNAs (**a**) and lncRNAs (**b**) detected by RNA-Seq. Transcripts with log_2_^f^°^ld change^ ≤ − 1 are shown in green, with a log_2_^f^°^ld change^ ≥ 1 in red, and with 1 > log_2_^f^°^ld change^ > − 1 in gray. **c** The Log_2_^TPM^ scatter plot depicting expression changes of miRNAs detected by miRNA-Seq. Transcripts with fold change ≤0.667 are green, those with fold change ≥1.5 are red, and others are gray

To identify the estrogen-responsive protein-coding genes and lncRNA genes in liver, RNA-Seq of livers from chickens treated with 17β-estradiol and the control groups was performed. An overview of the RNA-Seq data is presented in Table S1. After assembly, a total of 15,556 protein-coding genes were identified. Among them, 540 genes were significantly upregulated and 422 were significantly downregulated (|log_2_^f^°^ld change^| ≥ 1 and false discovery rate (FDR) ≤ 0.05; Fig. 2a; Table S2), including some known estrogen target genes, such as *APOB*, *APOV1*, *PPARG*, *LPL*, and *THRSP* [17, 19, 20]. The top five genes with the largest changes in the mRNA level were ENSGALG00000020772 (uncharacterized protein), ENSGALG00000012204 (uncharacterized protein), ENSGALG00000001863 (*VTG2*), ENSGALG00000015134 (*APOV1*), and ENSGALG00000008900 (*VTG1*). A total of 4144 known lncRNAs and 1598 novel lncRNAs were identified. Among them, seven lncRNAs were significantly upregulated and 10 lncRNAs were significantly downregulated (|log_2_^f^°^ld change^| ≥ 1 and FDR ≤ 0.05; Fig. 2b; Table S2).

To identify the estrogen-responsive miRNA genes, miRNA-Seq of the livers from the 17β-estradiol treated and the control chickens was performed. A total of 23.08 million reads per sample were generated (Table S3). In total, 575 known miRNAs and 153 novel miRNAs were identified. Among them, five miRNAs were significantly upregulated and six were significantly downregulated (fold change ≥1.5, and FDR ≤ 0.05; Fig. 2c; Table S2).

### Functional annotation of estrogen-responsive mRNA and ncRNA

To study the functions of the differentially expressed estrogen-responsive genes, Gene Ontology (GO) and Kyoto Encyclopedia of Genes and Genomes (KEGG) pathway enrichment analyses were performed. The GO enrichment analysis showed that the set of DE genes was significantly enriched in 59 GO terms. Among them, 17 (28.8%) GO terms were lipid-related (Table 1), including lipid localization (GO: 0010876), fatty acid metabolic process (GO: 0006631), lipid biosynthetic process (GO: 0008610), and lipid transport (GO: 0006869). KEGG pathway enrichment analysis indicated that the set of DE genes was significantly enriched in 14 KEGG pathways. Seven (50%) KEGG pathways were lipid-related (Table 2), which included fatty acid metabolism (gga01212), fatty acid degradation (gga00071), fatty acid biosynthesis (gga00061), and the PPAR signaling pathway (gga03320).

**Table 1:** The GO terms correlated to lipid metabolism

GO_ID	GO_term	Number of DE genes	Fold Enrichment	***q***-value
GO:0006629	lipid metabolic process	94	2.155	0.000
GO:0006631	fatty acid metabolic process	32	3.514	0.000
GO:0010876	lipid localization	29	2.899	0.001
GO:0055088	lipid homeostasis	15	4.773	0.001
GO:0008610	lipid biosynthetic process	46	2.234	0.001
GO:0006869	lipid transport	24	2.772	0.006
GO:0019395	fatty acid oxidation	11	4.288	0.015
GO:0016125	sterol metabolic process	12	3.742	0.020
GO:0016126	sterol biosynthetic process	7	6.420	0.021
GO:0034440	lipid oxidation	11	4.084	0.021
GO:0006639	acylglycerol metabolic process	11	3.898	0.023
GO:0006638	neutral lipid metabolic process	11	3.811	0.027
GO:0016042	lipid catabolic process	20	2.515	0.033
GO:0046464	acylglycerol catabolic process	5	7.796	0.041
GO:0050994	regulLation of lipid catabolic process	7	5.197	0.042
GO:0006633	fatty acid biosynthetic process	13	3.118	0.043
GO:0010883	regulLation of lipid storage	7	4.961	0.049

Fold Enrichment: fold enrichment = GeneRatio / BgRatio, the results were obtained by David tool

**Table 2:** The KEGG pathways correlated to lipid metabolism

Pathway ID	Pathway name	Different genes	*q*-value
gga03320	PPAR signaling pathway	*ACSBG2, FADS2, ACAA1, CPT1A, SCD, DBI, PLIN2, PPAR, FABP4, ACOX1, PLIN1, CYP7A1, ACSL1, PEPCK, LPL, EHHADH, LFABP, FABP3*	4.88E-06
gga01212	Fatty acid metabolism	*ACSBG2, FADS2, ACACA, ACAA1, CPT1A, SCD, ACAT2, ACOX1, FADS1, FASN, ELOVL2, ACSL1, ELOVL5, EHHADH, HSD17B12*	2.09E-05
gga00100	Steroid biosynthesis	*HSD17B7, FDFT1, DHCR24, LSS, SQLE, NSDHL, MSMO1, CYP51A1, DHCR7*	0.00013
gga01040	Biosynthesis of unsaturated fatty acids	*FADS2, ACAA1, SCD, ACOX1, FADS1, ELOVL2, ELOVL5, HSD17B12*	0.00232
gga00071	Fatty acid degradation	*ACSBG2, ACAA1, CPT1A, ECI2, ALDH3A2, ACAT2, ACOX1, ACSL1, EHHADH*	0.00328
gga00564	Glycerophospholipid metabolism	*PISD, ETNPPL, LPIN2, AGPAT4, PEMT, CHKA, GPAM, GNPAT, LYPLA1, AGPAT2, AGPAT9, LCAT, ETNK2, PHOSPHO1, MBOAT2*	0.00569
gga00061	Fatty acid biosynthesis	*ACSBG2, ACACA, FASN, ACSL1*	0.01620

**Fig. 3:**
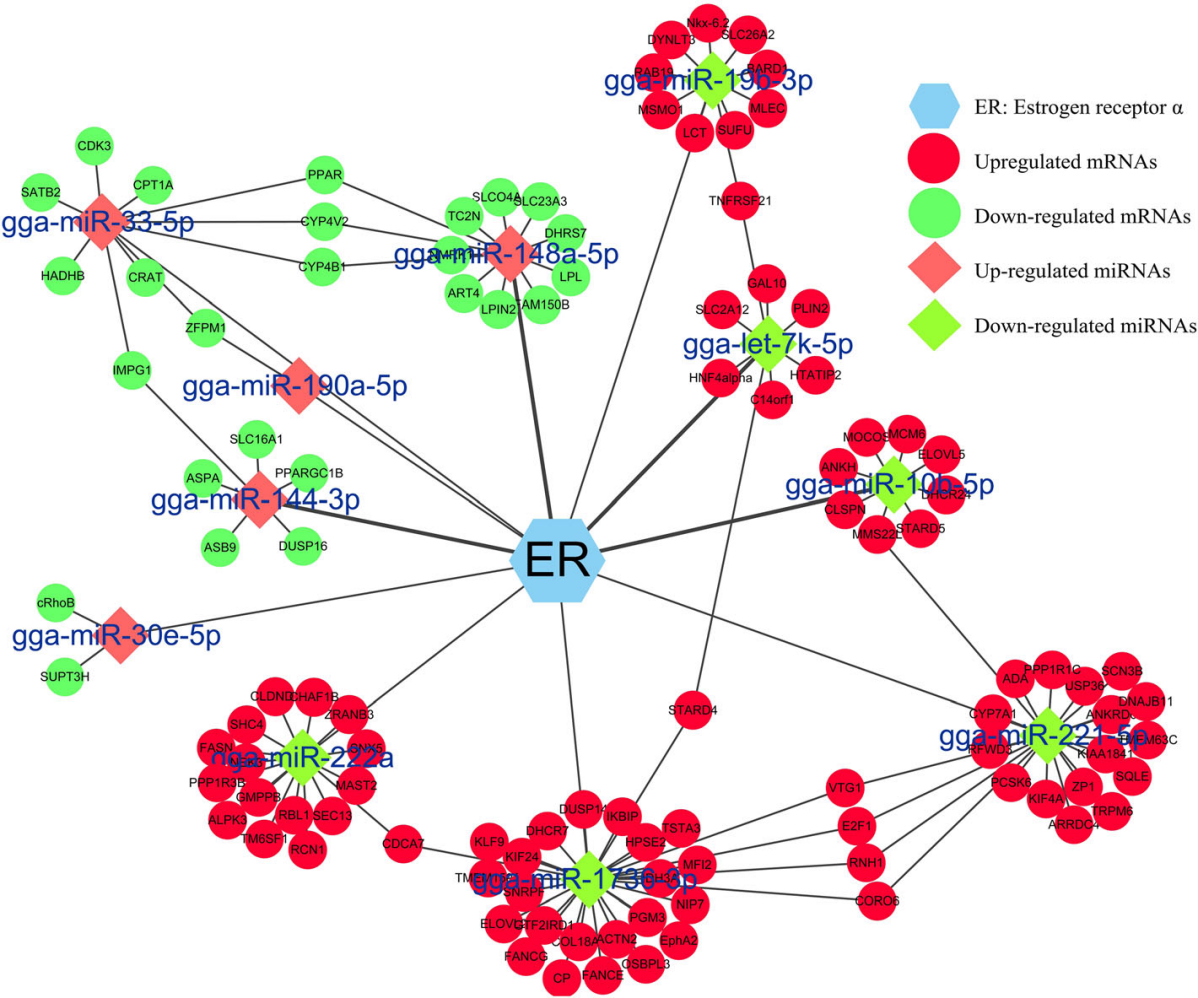
A network of estrogen-responsive miRNAs and their putative target genes. Circular nodes represent mRNAs whereas diamond nodes represent miRNAs. Red and green colors denote up- and downregulation, respectively

To assess the possible participation of miRNAs in lipid metabolism, the potential target genes of the 10 estrogen-responsive miRNAs were predicted by means of miRDB, TargetScan, and PicTar. There were 122 high-scoring potential miRNA-mRNA pairs among the 11 estrogen-responsive miRNA genes and 109 differentially expressed estrogen-responsive protein-coding genes. A network of the differential expressed miRNAs and predicted target genes was constructed using Cytoscape (Fig. 3). Function annotation of the 109 potential target genes showed that the gene set was significantly enriched in lipid-related terms, including lipid biosynthetic process (*p* = 1.47E-05), fatty acid metabolism pathway (*p* = 3.45E-05), and PPAR signaling pathway (*p* = 0.0001). These estrogen-responsive miRNA genes might be involved in hepatic lipid metabolism.

### Genome-wide identification of ERα binding sites

**Fig. 4:**
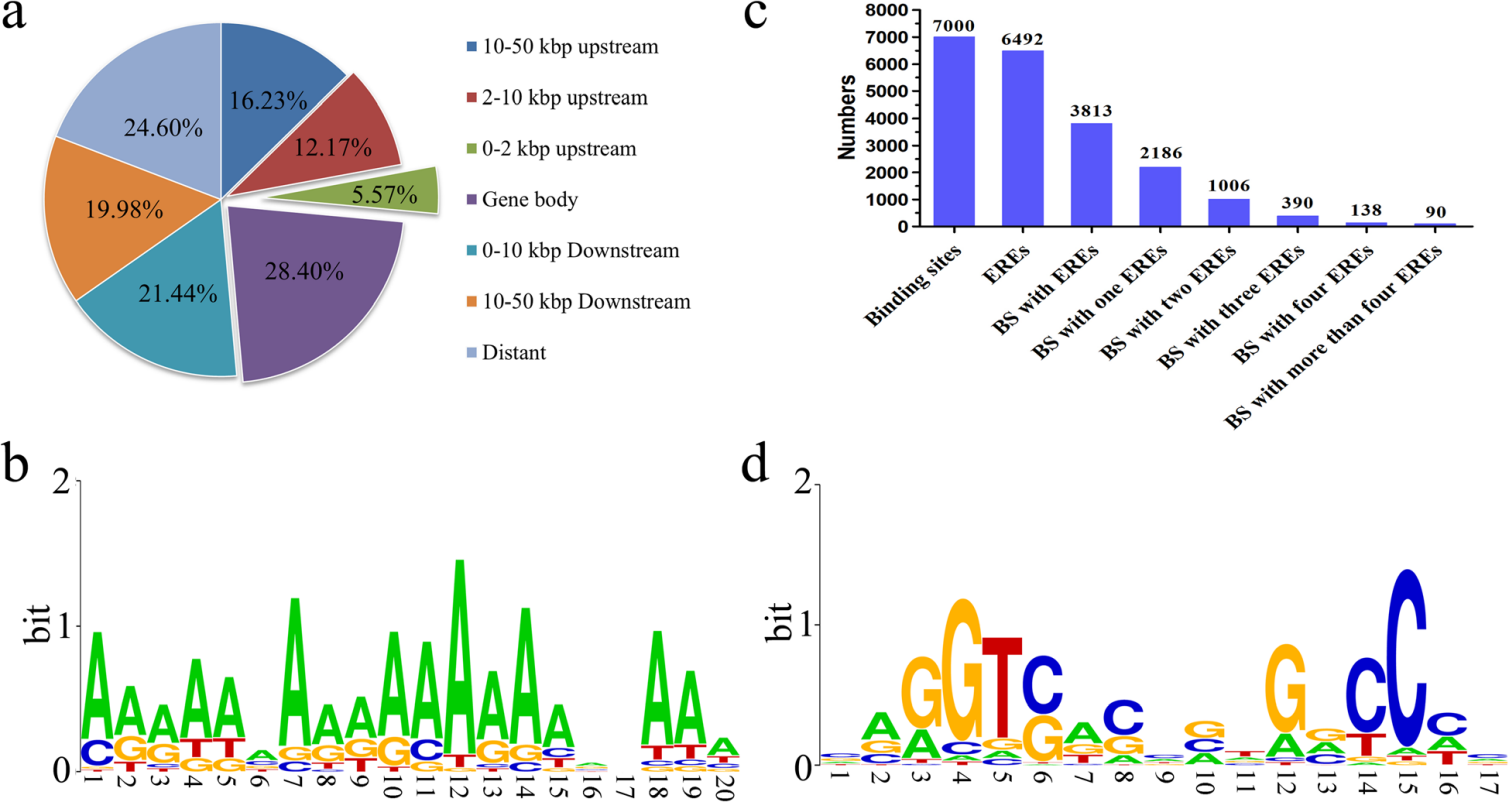
ChIP-Seq data analyses. **a** Genomic localization of called ERα-derived ChIP-Seq peaks. **b** Enrichment of the FOXP1 binding motif in the 500 bp ChIP signal sequence. **c** Distributions of the predicted EREs on the ERα-binding sites. “BS” stands for binding sites. The exact number is shown at the top of each column. **d** The ERE motif reconstructed by all the identified ERE sequences

To determine the estrogen-responsive mRNAs mediated via ERα binding sites, ChIP-Seq was conducted and a data summary is given in Table S4. A total of 7000 unique ERα binding sites were identified. We found that a low proportion (17.7%) of the binding sites were located < 10 kb upstream of the annotated TSS of genes (Fig. 4a), while 41.4% of the binding sites were located downstream of the annotated TSS of genes (Fig. 4a). All of the identified binding sites were used to perform an unbiased search for enriched motifs using MEME. The most significantly enriched motif was FOXP1 (*p* = 5.6e− 44) (Fig. 4b). FIMO was then used to identify ERE sequences in the identified binding sites. The count matrix motif of ERE was obtained from the JASPAR database (ID: MA0112.1). This analysis was performed with stringent criteria for ERE detection. The count matrix motif of an ERE was used, with a *p* value of < 0.0005 and a maximum of four mismatched residues allowed within the core 15 bp consensus ERE sequence (AGGTCAnnnTGACCT). Out of the 7000 unique ERα binding sites, 6492 putative EREs were identified (Fig. 4c). Only 3813 (54.5%) of the binding sites occupy the putative EREs. Approximately 47% of the ERα binding sites do not occupy any discernable EREs. A total of 42.7% of the binding sites that occupied ERE were more than one putative ERE (Fig. 4c). All of the identified ERE sequences were analyzed and a count matrix motif of an ERE was reconstructed (Fig. 4d). Notably, the trinucleotide spacer sequence NNN located in the middle of the ERE seems to be preferentially in the form of CNG. These findings suggest that the presence of a consensus ERE is not necessary for the binding of ERα to DNA in the chicken.

### Identification, functional analysis, and validation of the estrogen-responsive genes directly targeted by ERα via EREs

**Fig. 5:**
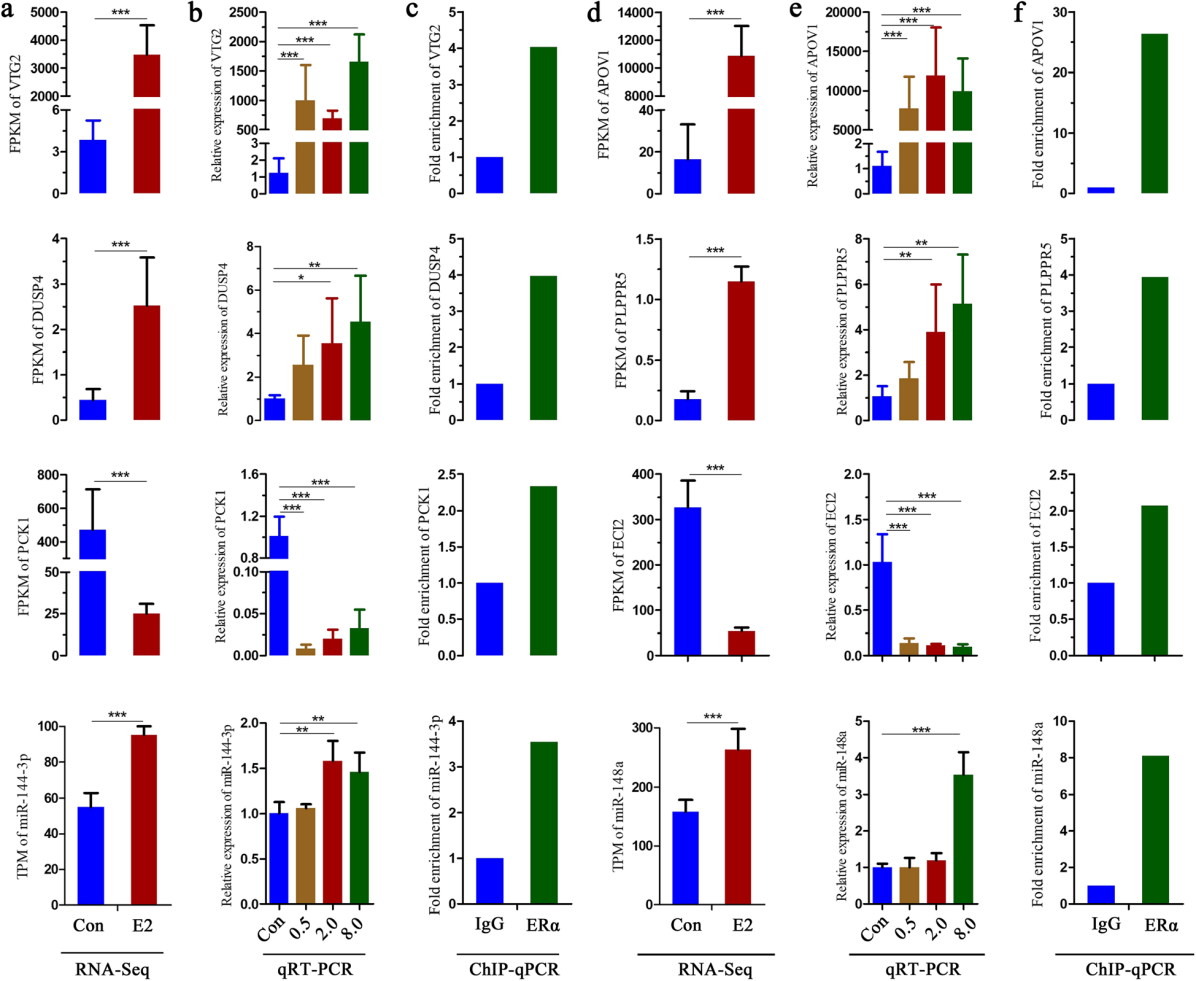
Confirmation of the identified genes directly targeted by estrogen. **a**, **d** FPKM/TPM data of the genes detected by RNA-Seq. **b**, **e** Effects of 17β-estradiol on the mRNA expression of the genes detected by qRT-PCR. Error bars represent SEM, *n* = 6 for the control group and *n* = 8 for the 17β-estradiol treatment groups, *p value < 0.05, **p value < 0.01, ***p value < 0.001. **c**, **f** Gene-specific ChIP-qPCR analysis. Chromatin was immunoenriched with an antibody to ERα or a control IgG

Estrogen-responsive genes that contain ER-binding sites < 50 kb upstream of a TSS were considered to be direct targets of estrogen. A total of 113 genes (11.5% of all estrogen-responsive genes) directly targeted by ERα via binding with EREs were identified (Table S5). Among those, four genes were miRNA, and the others were protein-coding genes. Eight genes among the 113 putative estrogen direct target genes were selected to perform qRT-PCR and ChIP-qPCR validation, including four randomly selected protein-coding genes *DUSP4*, *PLPPR5*, *PEPCK1*, and *ECI2*, two randomly selected miRNAs *miR-144-3p* and miR-148a, and two known classic estrogen target genes *VTG2* and *APOV1*. Our results proved that the expression of eight genes was significantly altered by estrogen (*P < 0.05*), and consistent with the RNA-Seq results (Fig. 5a, b, d and e). The ChIP-qPCR results showed that all eight binding sites were identified effectively (Fig. 5c, f).

### Estrogen suppresses PPARGC1B and DUSP16 expression by promoting miR-144-3p

**Fig. 6:**
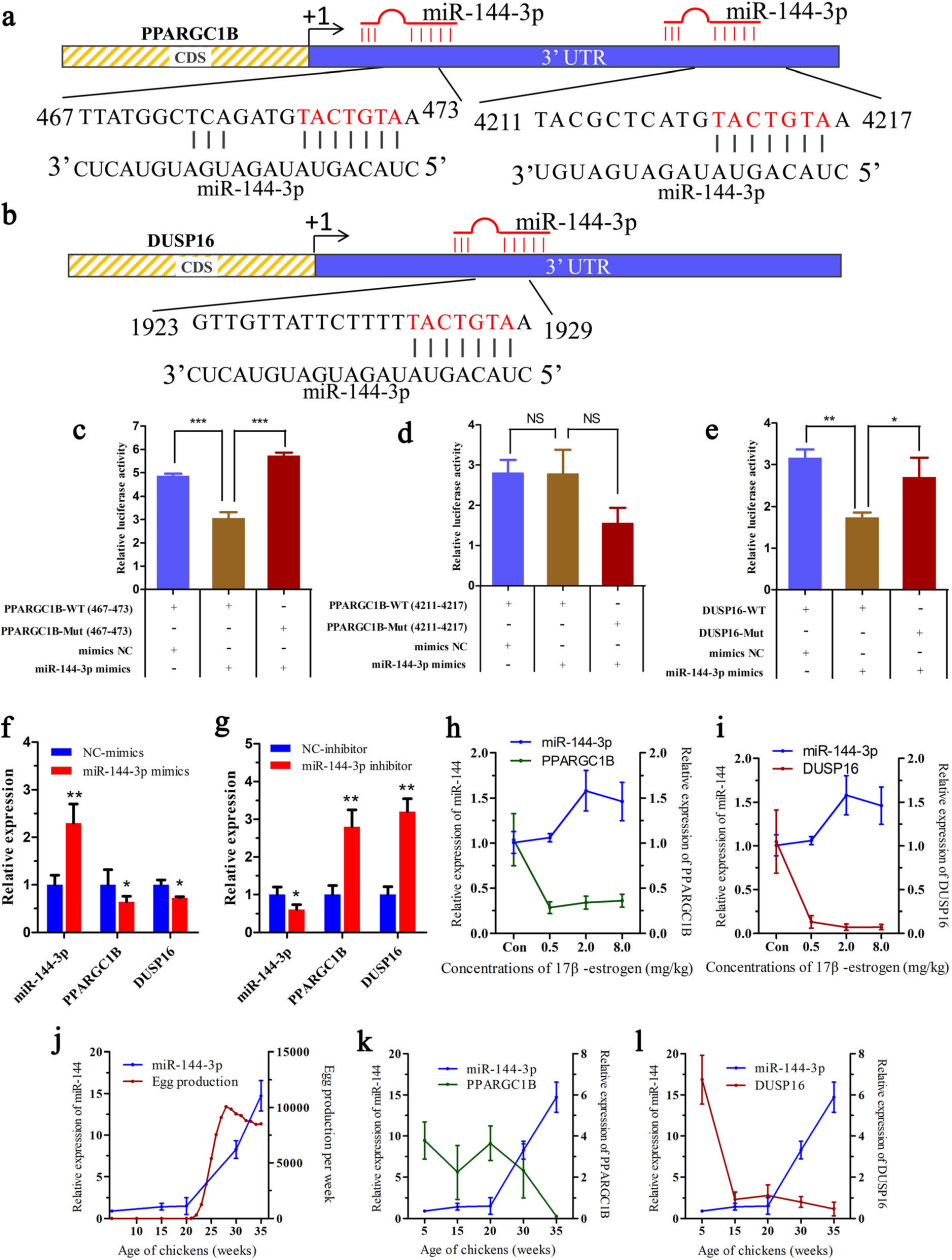
MiR-144-3p targets and suppresses the expression of *PPARGC1B* and *DUSP16* in the chicken liver. **a**, **b** The potential miR-144-3p binding sites in the 3′-UTR of *PPARGC1B* (**a**) and *DUSP16* (**b**). The exact location and the sequence of the binding sites are indicated. **c**–**e** Luciferase activities driven by the three miR-144-3p binding sites (~ 200 bp) in DF1 cells transfected with the miR-144-3p mimic or mimic NC (negative control). Luciferase activities were measured 48 h after transfection. Error bars represent SEM, *n* = 3, *p < 0.05, **p < 0.01. **f** The miR-144 mimic or mimic NC was transfected into chicken primary hepatocytes. After 24 h, qRT-PCR was performed to determine the expression levels of miR-144-3p, *PPARGC1B*, and *DUSP16*. **g** The miR-144-3p inhibitor or inhibitor NC was transfected into chicken primary hepatocytes. After 24 h, qRT-PCR assays were performed to determine the expression levels of miR-144-3p, *PPARGC1B*, and *DUSP16*. **h**, **i** Expression patterns of miR-144-3p and *DUSP16* and *PPARGC1B* mRNAs in response to 17β-estradiol in the chicken liver according to qRT-PCR analysis. All data are presented as mean ± SEM, n = 6 for the control group and *n* = 8 for the 17β-estradiol treatment groups. **j** Comparison between the expression curve of miR-144-3p and the egg production curve of the Lushi green-shelled-egg chickens. Each point in the egg production curve represents the cumulative egg production of 2000 chickens in one week. **k**, **l** Expression levels of miR-144-3p and *DUSP16* and *PPARGC1B* mRNAs in the chicken liver at different ages of chickens. Expression levels were determined by qRT-PCR. All data are presented as mean ± SEM

To confirm the reliability of the analysis results, the relationships among estrogen, gga-*miR-144-3p*, and the potential target genes *PPARGC1B* and *DUSP16* were verified. The three predicted binding sites of *miR-144-3p* in PPARGC1B and DUSP16 3′-UTR are shown in Fig. 6a, b. A dual-luciferase assay determined that *miR-144-3p* interacted with the site nucleotide positions 467-473 nt located in the 3’UTR of *PPARGC1B* and the site 1923–1929 nt located in the 3’UTR of *DUSP16* (Fig. 6c-e). Then, the *miR-144-3p* mimic and inhibitor were transfected into the chicken primary hepatocytes. The results indicated that overexpression of *miR-144-3p* could significantly suppress the mRNA expression of *PPARGC1B* and *DUSP16* (*P < 0.05*) (Fig. 6f), whereas knockdown of *miR-144-3p* significantly increased the mRNA expression of *PPARGC1B* and *DUSP16* (*P < 0.05*) (Fig. 6g). The 17β-estrogen treatments significantly downregulated *PPARGC1B* and *DUSP16* in chicken livers but upregulated *miR-144-3p* (*P < 0.05*) (Fig. 6h, i). Another data suggested a similar changing tend between the *miR-144-3p* expression and the egg production records of 2000 Lushi green-shelled-egg chickens (Fig. 6j). Moreover, the expression levels between *miR-144-3p* and *PPARGC1B*, *DUSP16* in the liver of chickens at different developmental stages presented a negative correlation (Fig. 6k, l). These findings indicate that estrogen suppresses the expression of *PPARGC1B* and *DUSP16* by promoting the expression of *miR-144-3p* in the chicken liver.

## Discussion

### Lipid metabolism in chicken liver is affected by estrogen level

Estrogen can strongly affect lipid metabolism in the liver and adipose tissue of mammals [1, 3]. We found 962 differentially expressed estrogen-responsive protein coding genes and 10 estrogen-responsive miRNAs were identified in the livers of 17β-estradiol treated hens. These included some known estrogen target genes and some novel estrogen-responsive genes, which are reported for the first time, e.g., *ENSGALG00000020772*, *ENSGALG00000012204*, and *ENSGALG0000002409*. Functional analysis showed that the set of estrogen-responsive genes, including the predicted target genes of miRNAs, were significantly enriched in steroid metabolism, steroid biosynthesis, lipid metabolism, lipid localization, and PPAR signaling. We also found, using miRNA-Seq analysis, that miR-148a, which is linked to lipid metabolism [21, 22], was one of the most abundant miRNAs in the chicken liver. Several other differentially expressed known miRNAs participate in lipid metabolism, including *miR33* [23], *miR-10b* [24], and *miR-30e* [25]. We previously reported a transcriptome profile of the liver of pre-laying and peak-laying chickens [6]. During the laying stage, the expression of lipid-related genes was highly stimulated in the liver to support the metabolic demands associated with reproductive organ development. Thus, most of the DEGs were considered to be lipid-related. GO and KEGG enrichment results demonstrated that DEGs were significantly enriched in lipid metabolism–related processes. We found that 33% of these genes are estrogen-responsive genes. The plasma estrogen level in female chickens reaches its peak before the onset of production of the first egg [7, 8], then drops gradually, but remains at a higher level than that in immature pullets [8]. Thus, we suggest that some liver DE genes between pre-laying and peak-laying chickens are estrogen inducible. The TG content in the serum of chickens was significantly increased after estrogen treatment, and the oil red O staining of liver tissue showed more lipid droplets in the livers of estrogen-treated chickens than in livers of the control group. Some estrogen-responsive genes are regulated during the laying period because of the increased plasma estrogen level, and they therefore participate in liver lipid metabolism. These findings provide details on the regulation of lipid metabolism by estrogen signaling in the chicken liver.

### Location relationships between ERα binding sites and estrogen-responsive genes

Estrogen exerts physiological functions mainly via the ERα and ERβ, which directly bind to the consensus EREs or indirectly bind to nuclear DNA by interacting with other transcription factors to regulate gene expression [9–11]. ERα is mainly expressed in the female reproductive system, liver, kidney, white adipose tissue and bone, while ERβ is expressed in ovary, lung, and other tissues [26]. In laying hens, more than 90% of de novo fatty acid synthesis occurs in the liver, rather than adipose tissue, kidneys, or the gastrointestinal tract. Therefore, the chicken liver is one of the best models to study lipid metabolism. In this study, a total of 7000 ERα binding sites and 113 of the 962 estrogen-responsive genes directly targeted by estrogen via ERα binding to the special ERE sequence were identified in the chicken genome by ChIP-Seq. Unexpectedly, most of the identified binding sites were located away from the annotated TSS and only a small proportion (~ 5%) of the binding sites were located < 2 kb upstream of a TSS. Similar results in humans have been reported [16, 27]. Furthermore, more than 60% of the binding sites were located in the gene body and downstream of genes. However, considering that many genes occupy alternative TSS, the binding sites that were located at a gene body may also be located at the promoter of one of the gene’s transcript isoforms. We do not clearly understand the induction/suppression of the transcription of genes by ERα, but our observations suggest the existence of long-range interactions between ERα binding sites and the regulated promoters. Our study revealed that approximately 50% of all ERα binding sites do not occupy a typical ERE or discernable ERE. A similar result was reported by Mason [28]. One reason for the absence of ERE sequences at many ERα binding sites is that ERα coupled with alternative transcription factors and thus bound to DNA targets via other transcription factors motifs. For example, the well demonstrated ER-FOXA1 complexes and ER-sp1 [11, 12, 29]. Therefore, understanding the cooperating factors associated with estrogen receptors will help uncover the relationship between the estrogen receptor and its responsive genes.

### Estrogen receptors interact with several cooperating factors

Estrogen regulates gene expression by two signaling pathways through ERα and ERβ. The canonical model for ER-mediated regulation of gene expression involves the direct binding of dimeric ER to EREs. In addition, ER can indirectly associate with promoters through protein-protein interactions with other DNA-binding transcription factors. A motif analysis for ERα binding sites throughout the human and mouse genomes revealed that other transcription factors generally cooperate with ERα and modulate estrogen receptor functions. For example, *FoxA1* [15, 30], *FOXP1* [29], and *AP1* [16] are important regulatory factors. In the present study, *FOXP1* was identified as the highest-confidence motif in extended ERα binding sites. Together with the high similarity between the identified ERE sequence and the ERE sequence reported in mammals, our findings suggest that the avian estrogen signaling pathway is similar to that of mammals. Although numerous interacting molecules at the genome scale were identified, high resolution of the complexes at one specific genomic locus remains lacking. Liu et al. described a CRISPR affinity CAPTURE approach to identify locus-specific chromatin-regulating protein complexes without bias [31]. Their method could be helpful for identifying the interacting molecules at specific ERE loci. It was also reported that, if signaling from ER-dependent AP1 elements occurs in estrogen target tissues, ERα and ERβ would respond differently to ligands at AP1 sites. This reveals a potential control mechanism for the transcriptional regulation of estrogen-responsive genes [9]. Together with the various cooperating factors enriched on ERα binding sites, estrogen is less dependent on ER, and ER is less dependent on an ERE than previously thought. However, ERα occurs in a ligand-independent manner.

### Non-coding RNAs involved in estrogen regulation network

Previous studies have focused on estrogen-responsive protein-coding genes. However, noncoding genes can have a variety of functions, especially the post-transcriptional regulation of gene expression or guidance of RNA modifications [32, 33]. Several studies have reported the expression profiles of miRNAs and lncRNAs after the administration of estrogen. A study on the estrogen-mediated miRNA expression profiles during cardiomyocyte hypertrophy demonstrated that estrogen can alter miRNA expression to inhibit cardiomyocyte hypertrophy [34]. Another study uncovered 34 DE miRNAs after 6 and 12 weeks of estrogen exposure, but only 6 differentially expressed miRNAs after 18 weeks of estrogen exposure [35]. A cohort of estrogen-responsive lncRNAs was identified using estrogen receptor–positive and –negative breast tumors [36]. These studies focused on the genes manifesting differential expression after estrogen administration, but the group of noncoding genes directly or indirectly regulated by estrogen has not been studied. We focused on the genome-wide identification of noncoding RNAs whose expression is directly and indirectly regulated by estrogen. Four noncoding genes were likely to be directly targeted by estrogen. The number of estrogen-responsive noncoding genes appears much lower than that of protein-coding genes. Nevertheless, because a single noncoding RNA can target multiple protein-coding genes [37], it is possible that these noncoding genes can participate in the estrogen signaling pathway at a genome-wide scale. One of the estrogen-responsive miRNAs, miR-148a, can target more than 10 genes, such as *MSK1* [38] and *P27* [39]. Hundreds of estrogen-responsive genes were demonstrated to be putative target genes of these estrogen-responsive miRNAs. These results increase our understanding of the non-coding RNA mediated estrogen regulation network in the liver of chickens.

### *miR-144* mediated lipid metabolism regulation by estrogen

**Fig. 7:**
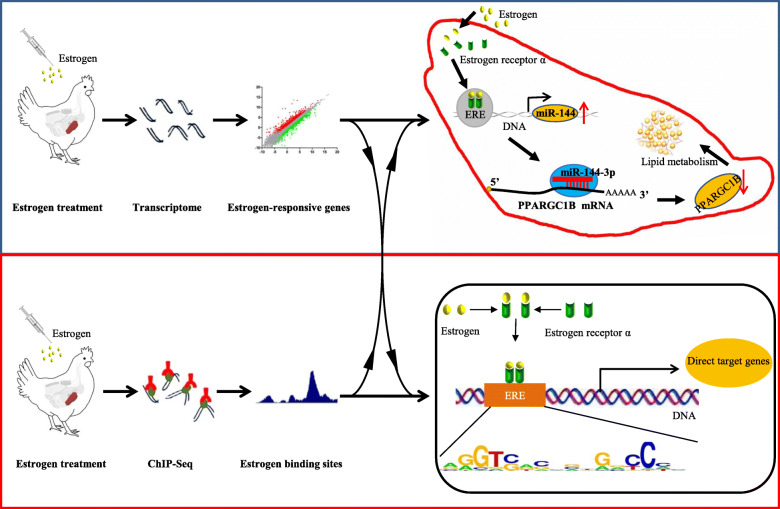
Model of estrogen-mediated lipid metabolism by miR-144 and estrogen functional sites

Both *miR-144* and *PPARGC1B* are reported to play important roles in lipid metabolism and The *miR-144* is regulated by estrogen [40]. However, no detailed network has been constructed to describe miR-144-mediated lipid metabolism. In this study, we demonstrated that the *miR-144-3p* gene is directly targeted by estrogen via ERα. The upregulated *miR-144-3p* suppresses the expression of its target gene *PPARGC1B* by binding to the 3’UTR of *PPARGC1B* mRNA. Downregulation of *PPARGC1B* therefore changes the lipid metabolism in the chicken liver (Fig. 7). We previously demonstrated that estrogen could repress the expression of *gga-miR-221-5p* and increase the expression of targets *ELOVL6* and *SQLE* to promote lipid synthesis in chicken liver [41]. Thus, identification of noncoding genes targeted by estrogen is helpful for understanding the mechanism of the estrogen signaling pathway.

## Conclusions

In conclusion, our integrated analysis of RNA-Seq data and ChIP-Seq data identified estrogen receptor functional sites in the chicken genome and all of the genes, including protein-coding genes, miRNAs and lncRNAs, regulated via estrogen in the liver of chicken (Fig. 7). We also demonstrated the functional role of estrogen-responsive genes in lipid metabolism. Estrogen directly targeted *miR-144-3p* mediated lipid metabolism by suppressing the *PPARGC1B* expression level (Fig. 7). These results increase our understanding of the functional network regulated by estrogen in liver of chicken and provide insight into estrogen mediated lipid metabolism.

## Methods

### Animals and sampling

All animals used in the experiments were female Lushi blue-shell-egg chickens obtained from the Animal Center of Henan Agricultural University. The chickens were housed in cages separately under the same environmental conditions with ad libitum access to food and water. The corn soybean basal diets containing 15% crude protein, 5% crude fat, 6% Crude Fiber, and 2750 kcal/kg energy were obtained from Shandong Newhope Liuhe Group Co., Ltd. (Liuhe, Shandong, China). A total of 30 chickens with similar body weight (680 ± 24.6 g) at 10 weeks of age were selected and subdivided randomly into four groups. Three groups (*n* = 8) were injected intramuscularly with 17β-estradiol (dissolved in olive oil) (Sigma, St. Louis, MO, USA) at 0.5, 2.0 or 8.0 mg / kg of body weight. One group (*n* = 6) serving as a control was given vehicle (olive oil) only. All chickens were euthanized by cervical dislocation at 12 h after treatment. A portion of the fresh liver tissue was embedded with optimum cutting temperature compound for oil red O staining. The rest of the liver was snap-frozen in liquid nitrogen and stored at − 80 °C in a freezer until use. Three livers from the control group and three livers from the 8.0 mg/kg 17β-estradiol group were subjected to RNA-Seq. Livers from the 8.0 mg/kg 17β-estradiol group were also used for ChIP-Seq.

### Primary hepatocytes culture and estrogen administration

The isolation, purification, and culture of chicken primary hepatocytes were performed as described previously [19, 42]. The chicken hepatocytes were isolated from 18-day chicken embryonic livers. Cells were divided into four groups with triplicates. The cells were starved for 6 h after they grew to 80% confluence. Then, we added 17β-estradiol dissolved in 0.1% ethanol to final concentrations of 25 nM, 50 nM, or 100 nM. Cells treated with ethanol only were used as controls. After 12 h incubation, cells were collected and stored at − 80 °C until use.

### Oil red O staining

The optimum cutting temperature compound-embedded liver tissue sample was sectioned with a freezing microtome. The sections were washed with PBS, fixed with 4% paraformaldehyde for 30 min at room temperature and then stained with 0.2% oil red O solution (Sigma) for 15 min. After staining, sections were washed with PBS and counterstained with hematoxylin for 5 min. The washed sections were observed under a microscope at 200× and 400× magnifications.

### RNA-Seq

Total RNA was extracted from the chicken liver tissues using the TRIzol® reagent following manufacturer instructions (Invitrogen, Carlsbad, CA, USA). Libraries were constructed using the TruSeq Stranded Total RNA LT (with Ribo-Zero™ Gold) Set B (cat. # RS-122-2302; Illumina, San Diego, CA, USA). The libraries were sequenced on the Illumina HiSeq 4000 platform via the 150 bp pair-end sequencing strategy following manufacturer instructions. The obtained raw reads were cleaned using the FASTX-Toolkit (http://hannonlab.cshl.edu/fastx_toolkit/index.html). FASTQ files of clean paired-end reads were aligned to the reference genome using TopHat. The reference genome for the chicken (*Gallus* 5.0) was downloaded from the UCSC Genome Browser. The transcript abundance and putative novel mRNA isoforms were analyzed in the Cufflinks software. Next, the FPKM values were used to quantify the gene expression levels. In this study, the FDR was used to determine the threshold of the *p* value in multiple tests and analyses.

After annotation, the unannotated transcripts were employed to identify lncRNAs. We applied the following filtering criteria: (1) exon number ≥ 2; (2) transcript length ≥ 200 bp; (3) predicted open reading frame < 300 bp; (4) the transcript aligned to Pfam [43] without significant hits; (5) coding–noncoding index score < 0 [44]; and (6) coding potential calculator score < 0 [45].

### miRNA-Seq

Small RNA was extracted from chicken liver tissue samples using the mirVana™ miRNA Isolation Kit (cat. # AM1561, Ambion, Austin, TX, USA). The extracted RNA samples were ligated sequentially with 3′ and 5′ RNA adapters by means of T4 RNA ligase (cat. # M0242l; BioLabs, Beverly, MA, USA). The ligated RNA samples were reverse-transcribed and amplified by PCR to generate cDNAs. The cDNAs of appropriate lengths were purified from an agarose gel to construct sequence libraries. After purification, the small-RNA libraries were quantified on a Qubit Fluorometer (Invitrogen) and used for cluster generation and 50 bp single-end sequencing analysis with an Illumina HiSeq 2000 system.

An initial filtering step was performed using the Fastx-toolkit to remove adaptor sequences, low-quality reads (base quality less than 10), and short reads (shorter than 18 nt) before the clean reads were collected and subjected to bioinformatics analysis. Clean reads were first compared with the miRBase database 21.0 using the CLC Genomics Workbench 5.5 commercial software (CLC Bio, Aarhus, Denmark) to identify known miRNAs. The unmatched sequences were screened against the noncoding RNA database, Rfam, and piRNA database to filter out rRNAs, tRNAs, snRNAs, and snoRNAs. After the elimination of repeat-associated small RNAs, degradation fragments of mRNAs, and known miRNAs, the remaining reads were mapped to the chicken genome using Burrows–Wheeler Alignment to obtain pre-miRNA sequences [46]. The mapped sequences were then utilized to predict novel miRNAs by means of MIREAP. The expression of the identified miRNAs was normalized by calculation of TPM. The differential significance was identified using EdgeR software with the following thresholds: FDR < 0.05 and fold change ≥1.5 or fold change ≤0.667.

### ChIP-Seq

The ChIP assay was performed as described previously [19]. In brief, fresh liver tissue samples collected from chickens treated with 17β-estradiol were cross-linked with 1% formaldehyde, after which the cross-linking was stopped by the addition of 2.0 M glycine. Isolated DNA was fragmented and incubated with a monoclonal specific ERα antibody (0.2 mg/mL, cat. # MA5–13065; Invitrogen) or IgG antibody (Cell Signaling Technology, Danvers, MA, USA). The immunoprecipitated DNA fragments were quantified and used to construct the DNA libraries using the ChIP-Seq Sample Prep Kit (Illumina). The libraries were then sequenced on the Illumina HiSeq 2500 platform using 50 bp single-end sequencing analysis. Raw reads were subjected to a quality check and trimming, after which the obtained clean reads were aligned to the chicken genome (*Gallus* 5.0) via Burrows–Wheeler Alignment. Peak calling procedures were performed in MACS2 [47] with threshold *p* values ≤0.005. To perform a motif search, all positive peaks were used, and the peak sequences were extended on both sides to obtain a 500 bp sequence. The extended 500 bp sequences were then used to discover motifs using MEME and FIMO. MAST was employed for motif alignment to exclude duplicate motifs, whereas Tomtom was used for annotation of the discovered motifs.

### Functional enrichment

GO and Kyoto Encyclopedia of KEGG pathway enrichment analyses of genes were performed by DAVID. *p* value < 0.05 was used as the cut-off criterion for GO and KEGG pathway enrichment analyses.

### miRNA target gene prediction

The miRNA target prediction software programs miRDB, TargetScan 7.1 and PicTar were used to predict miRNA target genes. Only when miRNA-mRNA pairs were positively predicted by > 2 of these software programs were the pairs taken as a positive result.

### Luciferase assays

A target fragment was amplified by PCR with special primers containing XhoI and NotI restriction sites. Seven bases of binding sites in 3’UTR were deleted to create a mutant version by means of special primers designed for overlap extension PCR. Then, the PCR product was cloned into the XhoI-and-NotI double-digested psi-CHECK™-2 plasmid (Promega, Madison, WI, USA) by means of T4 ligase (Biolabs, Beverly, MA, USA). All of the constructed vectors were confirmed by PCR and sequencing (BGI, Shenzhen, China). A chicken embryonic fibroblast cell line (DF1) was cultured and cotransfected with the constructed luciferase vector and a miRNA mimic or mimic negative control (mimic NC) with Lipofectamine™ 2000 (Thermo, Waltham, MA, USA). The cell lysates were harvested 48 h after transfection. The *Renilla* luciferase and firefly luciferase activities were measured using a Dual Luciferase Reporter Assay System (Promega). For each transfected group, the procedure was performed in triplicate in at least three independent experiments.

### Quantitative real-time PCR (qRT-PCR)

The expression levels of some selected mRNAs and lncRNAs were validated by qRT-PCR. The PrimeScript™ RT Reagent kit with gDNA Eraser (TaKaRa, Dalian, China) was used to synthesize the cDNA according to manufacturer instructions. The qRT-PCR was performed using the SYBR Green method in a LightCycler® 96 instrument. Each reaction contained 5 μL of SYBR Green PCR Master Mix (TaKaRa), 3.5 μL of RNase-free water, 0.5 μL each of forward and reverse primers, and 0.5 μL of extracted cDNA. The reactions were amplified using the following conditions: denaturation at 95 °C for 5 min; followed by 40 PCR cycles at 95 °C for 30 s, 60 °C for 30 s, and 72 °C for 20 s; and then a further 10-min extension at 72 °C. All reactions were performed in triplicate. The expression levels were measured in terms of the cycle threshold (Ct) and then normalized to the expression of *β-actin* using the 2^−△△Ct^ method. The primers used were designed using the NCBI Primer-BLAST tool and synthesized by Sangon Biotech (Shanghai, China). All primer sequences are listed in Table S6.

Expression of the miRNA was detected by stem-loop qRT-PCR. Reverse transcription of miRNAs was performed using miRNA-specific stem-loop primers and the PrimeScript RT Reagent Kit (TaKaRa). The primers used for reverse transcription and qRT-PCR were designed and purchased from GenePharma Co., Ltd. (Shanghai, China). The miRNA expression levels were normalized to the expression of U6, and the other protocols were the same as described above.

### ChIP-quantitative PCR (ChIP-qPCR) analysis

The immunoprecipitated DNA fragments were also used to verify the interaction of ERα and selected binding sites by ChIP-qPCR. Meanwhile, 20% of starting chromatin without chromatin immunoprecipitation served as input to represent the unselected DNA content. The fold enrichment method was chosen to normalize the ChIP-qPCR data: Fold enrichment = log_2_^−ΔΔCt^, ΔCt = Ct (IP) − Ct (Input) − log_2_^5^, ΔΔCt = ΔCt − ΔCt (IgG). Gene-specific primers for the putative binding-site regions were designed using the NCBI Primer-BLAST tool and synthesized by Sangon Biotech (Shanghai, China). All ChIP-qPCR primer sequences used are listed in Table S7.

### Statistical analyses

Statistical analyses were carried out using SPSS version 20.0 (IBM, Chicago, IL, USA). One-way ANOVA were used for statistical analysis, followed by Dunnett’s test. The results were presented as Mean ± SEM of more than 6 replicates, *p* < 0.05 was considered statistically significant.

## Supplementary Information

**Additional file 1:** **Table S1.** Descriptive summary of data generated by RNA-seq.

**Additional file 2:** **Table S2.** List of estrogen-responsive genes.

**Additional file 3:** **Table S3.** Descriptive summary of data generated by miRNA-seq.

**Additional file 4:** **Table S4.** Descriptive summary of data generated by ChIP-seq.

**Additional file 5:** **Table S5.** List of genes directly targeted by estrogen.

**Additional file 6:** **Table S6.** qRT-PCR primers.

**Additional file 7:** **Table S7.** ChIP-qPCR primers.

## Data Availability

All data generated in this manuscript have been deposited in NCBI Sequence Read Archive under the accession number PRJNA434773.

## Abbreviations

VLDL: Very low-density lipoprotein
ERs: Estrogen receptors
EREs: Estrogen response elements
DE: Differentially expressed
3’UTRs: 3′-untranslated regions
miRNAs: microRNAs
lncRNAs: Long noncoding RNAs
FPKM: Fragment per kilobase of exon model per million mapped reads
FDR: False discovery rate
TPM: Transcripts per million clean reads
GO: Gene Ontology
KEGG: Kyoto Encyclopedia of Genes and Genomes
DF1: Fibroblast cell line
qRT-PCR: Quantitative real-time PCR
Ct: Cycle threshold
ChIP-qPCR: ChIP-quantitative PCR
APOB: Apolipoprotein B
APOV1: Apovitellenin 1
TG: Triglyceride
TSSs: Transcription start sites
