# Supplementary material for: Global investigation of estrogen-responsive genes regulating lipid metabolism in the liver of laying hens
Source: BMC Genomics. 2021 Jun 9;22:428. doi: 10.1186/s12864-021-07679-y (PMC8190866; doi:10.1186/s12864-021-07679-y)
Supplement: Supplementary file 1 — Additional file 1: Table S1. Descriptive summary of data generated by RNA-seq. [file 12864_2021_7679_MOESM1_ESM.docx]

Table S1 Descriptive summary of data generated by RNA-seq

| Sample ID | Raw reads | Clean reads | Clean ratio | Mapped reads | Mapping ratio |
| --- | --- | --- | --- | --- | --- |
| L10_1 | 128,536,770 | 100,418,845 | 78.1% | 83,837,034 | 83.8% |
| L10_2 | 132,976,370 | 104,770,029 | 78.8% | 86,978,300 | 83.4% |
| L10_3 | 117,182,044 | 89,497,410 | 76.4% | 74,506,328 | 83.5% |
| L10E_1 | 164,048,280 | 129,761,711 | 79.1% | 108,365,534 | 83.8% |
| L10E_2 | 175,971,864 | 134,819,648 | 76.6% | 111,526,393 | 83.1% |
| L10E_3 | 164,712,076 | 125,993,571 | 76.5% | 103,652,443 | 82.9% |
