# Supplementary material for: Global investigation of estrogen-responsive genes regulating lipid metabolism in the liver of laying hens
Source: BMC Genomics. 2021 Jun 9;22:428. doi: 10.1186/s12864-021-07679-y (PMC8190866; doi:10.1186/s12864-021-07679-y)
Supplement: Supplementary file 3 — Additional file 3: Table S3. Descriptive summary of data generated by miRNA-seq. [file 12864_2021_7679_MOESM3_ESM.docx]

Table S3 Descriptive summary of data generated by miRNA-seq

| Sample | Raw Reads | Effective reads | Effective ratio |
| --- | --- | --- | --- |
| L10_1 | 21,581,833 | 20,875,829 | 96.73% |
| L10_2 | 19,874,211 | 18,978,791 | 95.49% |
| L10_3 | 23,224,147 | 22,495,979 | 96.86% |
| L10E_1 | 24,356,179 | 24,006,261 | 98.56% |
| L10E_2 | 23,210,219 | 22,810,167 | 98.28% |
| L10E_3 | 26,224,095 | 25,825,270 | 98.48% |
