# Supplementary material for: Global investigation of estrogen-responsive genes regulating lipid metabolism in the liver of laying hens
Source: BMC Genomics. 2021 Jun 9;22:428. doi: 10.1186/s12864-021-07679-y (PMC8190866; doi:10.1186/s12864-021-07679-y)
Supplement: Supplementary file 4 — Additional file 4: Table S4. Descriptive summary of data generated by ChIP-seq. [file 12864_2021_7679_MOESM4_ESM.docx]

Table S4 Descriptive summary of data generated by ChIP-seq

| Sample | Raw reads | Clean reads | Clean ratio | Unique mapped | Mapping ratio |
| --- | --- | --- | --- | --- | --- |
| IP_1 | 25806669 | 25648644 | 99.39% | 21400608 | 84.58% |
| Input | 31467876 | 31238852 | 99.27% | 26044297 | 84.28% |
