# Supplementary material for: Global investigation of estrogen-responsive genes regulating lipid metabolism in the liver of laying hens
Source: BMC Genomics. 2021 Jun 9;22:428. doi: 10.1186/s12864-021-07679-y (PMC8190866; doi:10.1186/s12864-021-07679-y)
Supplement: Supplementary file 6 — Additional file 6: Table S6. qRT-PCR primers. [file 12864_2021_7679_MOESM6_ESM.docx]

Table S6 qRT-PCR primers

| **Gene** | **GenBank Accession** | **Primer sequence (5'-3')** | | **Product size** |
| --- | --- | --- | --- | --- |
| *APOV1* | NM_205483.2 | F: | CAATGAAACGGCTAGACTCA | 108 |
|  |  | R: | AACACCGACTTTTCTTCCAA |  |
| *VTG2* | NM_001031276.1 | F: | GAAGCTCTTCAGCCCATCCAT | 110 |
|  |  | R: | CTGGCTGGTTCTCCCATGTTA |  |
| *PCK1* | NM_205471.1 | F: | CAACACCAGATTCCCAGGCT | 81 |
|  |  | R: | AAAGGAGATCCAATCGGCCC |  |
| *DUSP4* | NM_204838.1 | F: | CCCTGGATTTGGGCTTCAGT | 231 |
|  |  | R: | TGTCGGCTTTGTGGTTGTCT |  |
| *ECI2* | XM_015276007.1 | F: | AAAGCCAAATGGGATGCGTG | 160 |
|  |  | R: | GGGTTTCATAGCCACCACGA |  |
| *PLPPR5* | XM_422320.5 | F: | TAAATCCACTGGTGCGCAGA | 194 |
|  |  | R: | GGCGTGTGCACTACTGATGA |  |
| *DUSP16* | XM_015290885.1 | F: | TGAAGCTTACAGATTTGTGAAAGAA | 219 |
|  |  | R: | GTGATGCAGAGCTGACTCGT |  |
| *PPARGCIB* | XM_015293715.1 | F: | TCCCTGCAAATGGGAACCTC | 186 |
|  |  | R: | GAGAGGGACAATGAGCCGTC |  |
| *β-actin* | NM_205518.1 | F: | GAGAGAAGATGACACAGATC | 116 |
|  |  | R: | GTCCATCACAATACCAGTGG |  |
