# Supplementary material for: Global investigation of estrogen-responsive genes regulating lipid metabolism in the liver of laying hens
Source: BMC Genomics. 2021 Jun 9;22:428. doi: 10.1186/s12864-021-07679-y (PMC8190866; doi:10.1186/s12864-021-07679-y)
Supplement: Supplementary file 7 — Additional file 7: Table S7. ChIP-qPCR primers. [file 12864_2021_7679_MOESM7_ESM.docx]

Table S7 ChIP-qPCR primers

| **Gene** | **GenBank accession** |  | **Primer sequence (5'-3')** | **Product size** |
| --- | --- | --- | --- | --- |
| *APOV1* | NM_205483.2 | F: | CCATTACCAAATCCGAACA | 156 |
|  |  | R: | CATCTGAGACACTGGCATTT |  |
| *VTG2* | NM_001031276.1 | F: | CCCTCTTGCCTTACTGCTGA | 152 |
|  |  | R: | GTTCTTTCAGCTCCGGTCAC |  |
| *PCK1* | NM_205471.1 | F: | GACTCCCTGCTTCCTACC | 146 |
|  |  | R: | GCACAGCCAATAACACCC |  |
| *DUSP4* | NM_204838.1 | F: | GGATGCAGTGTGTGCTAAA | 149 |
|  |  | R: | AGCTAAGGGAGTGAATGCC |  |
| *ECI2* | XM_015276007.1 | F: | AGGAACAGGACAGAAGTAT | 131 |
|  |  | R: | AACAAATGACAGAAAGAGC |  |
| *PLPPR5* | NM_205483.2 | F: | TTCCCCGTTGAACTGTGAG | 117 |
|  |  | R: | TTAGAACCCAAAGGATAAAGGAG |  |
| *miR-144* | NR_031585.1 | F: | AGGAAAGCGTGTACTGTGA | 132 |
|  |  | R: | TGTCGAACACAAACTCCTT |  |
| *miR-148a* | NR_031413.1 | F: | GTATCCCGCCAGGTAGCA | 153 |
|  |  | R: | GGGAGGACTGGAGGCACT |  |
